# Fanconi anemia protein FANCD2 is activated by AICAR, a modulator of AMPK and cellular energy metabolism

**DOI:** 10.1002/2211-5463.12185

**Published:** 2017-01-09

**Authors:** Min Jeong Chun, Hana Choi, Dong Wha Jun, Sunshin Kim, Yong‐Nyun Kim, Soo‐Youl Kim, Chang‐Hun Lee

**Affiliations:** ^1^ Cancer Cell and Molecular Biology BranchResearch InstituteNational Cancer CenterGoyangKorea; ^2^ Precision Medicine BranchResearch InstituteNational Cancer CenterGoyangKorea; ^3^ Comparative Biomedicine Research BranchResearch InstituteNational Cancer CenterGoyangKorea

**Keywords:** AICAR, AMP‐activated protein kinase, FANCD2

## Abstract

FANCD2 is a pivotal molecule in the pathogenesis of Fanconi anemia (FA), an autosomal recessive human syndrome with diverse clinical phenotypes, including cancer predisposition, short stature, and hematological abnormalities. In our previous study, we detected the functional association of FANC proteins, whose mutations are responsible for the onset of FA, with AMPK in response to DNA interstrand crosslinking lesions. Because AMPK is well known as a critical sensing molecule for cellular energy levels, we checked whether FANCD2 activation occurs after treatments affecting AMPK and/or cellular energy status. Among the treatments tested, AMPK‐activating 5‐aminoimidazole‐4‐carboxamide‐ribonucleoside (AICAR) induced monoubiquitination and nuclear foci formation of FANCD2, which are biomarkers of FANCD2 activation. FANCD2 activation was abolished by treatments with Compound C, an AMPK inhibitor, or after AMPKα1 knockdown, substantiating the involvement of AMPK in AICAR‐induced FANCD2 activation. Similarly, FANCA protein, which is a component of the FA core complex monoubiquitinating FANCD2, was required for this event. Furthermore, FANCD2 repression enhanced cell death upon AICAR treatments in transformed fibroblasts and cell cycle arrest in the renal cell carcinoma cell line Caki‐1. Overall, this study showed FANCD2 involvement in response to AICAR, a chemical modulating cellular energy metabolism.

**Abbreviations**
AICAR: 5‐aminoimidazole‐4‐carboxamide‐ribonucleoside
FA: Fanconi anemia
FANCD2: FA complementation group protein D2

Fanconi anemia (FA) is an autosomal recessive human syndrome with diverse phenotypes of short stature, congenital abnormalities, hematological disorders, cancer predisposition, and hypersensitivity to DNA crosslinking agents such as cisplatin and mitomycin C (MMC) 1. FA is caused by mutations in genes encoding the FANC proteins. So far, 21 FANC proteins have been identified including FANCU and FANCV 2, 3, and named as FANCA, FANCB, FANCC, and so on 4. Eight FANC proteins including FANCA and FANCL form the FA core complex that is activated by DNA‐damaging agents and FANCL E3 ligase, causing monoubiquitination of the FANCD2 protein 5, 6, 7. Monoubiquitinated FANCD2 localizes in the region of DNA damage, forming nuclear foci, and participates in the process of homologous recombinational DNA damage repair along with BRCA1 8, 9.

With regard to FA phenotypes, FA patients are prone to glucose/insulin abnormalities, such as glucose intolerance, hyperinsulinism, and diabetes mellitus 10. Recent studies have proposed the involvement of FA proteins in metabolic pathways and mitochondrial function. Several studies showed that FANCA‐deficient cells have damaged mitochondria and defects in mitochondrial respiratory chains 11, 12, 13. Another study showed that FANC proteins, including FANCA and FANCD2, are required for special autophagic processes of virophagy and mitophagy 14.

We have observed the functional association between FANC proteins and energy‐sensing AMP‐activated protein kinase (AMPK) 15. Based on the previous finding, we investigated whether FANC proteins have a role in cellular response to metabolic stress. AMPK is a well‐known energy sensor protein and is activated by high AMP and low ATP levels 16. We examined the effects of 5‐aminoimidazole‐4‐carboxamide‐ribonucleoside (AICAR) treatment on FANCD2 activation. AICAR is phosphorylated by adenosine kinase into 5‐amino‐4‐imidazolecarboxamide ribotide (ZMP), which is an AMP mimetic and directly activates AMPK 17, 18. Here, we present data supporting that AICAR treatment induces FANCD2 monoubiquitination and nuclear foci formation. AMPK was required for this FANCD2 activation. Furthermore, FANCD2 disturbance in normal fibroblasts and renal cell carcinoma (RCC) cells led to changes in AICAR‐induced cell cycle arrest and apoptosis.

## Materials and methods

### Cells

Transformed FANCA^−/−^ fibroblast (GM06914B), FANCD2^−/−^ fibroblast (GM16633A), and normal fibroblast (GM00637I) cell lines were obtained from the Coriell Institute for Medical Research in Camden, NJ, USA. Transformed fibroblast cell lines were grown in Dulbecco's modified Eagle's medium (DMEM) supplemented with 10% fetal bovine serum (FBS; GE Healthcare Life Sciences Hyclone Laboratories, Logan, UT, USA) at 37 °C in a humidified atmosphere containing 5% CO_2_. Caki‐1 RCC cell line was obtained from National Cancer Institute in Bethesda, MD, USA (NCI; MTA no. 270209) and was maintained in Roswell Park Memorial Institute 1640 (RPMI‐1640; GE Healthcare Life Sciences Hyclone Laboratories) supplemented with 10% FBS in a humidified atmosphere containing 5% CO_2_ at 37 °C.

### Treatments of chemicals

Cells were treated with energy stress‐inducing chemicals such as AICAR (Calbiochem, San Diego, CA, USA), 2‐deoxyglucose (Calbiochem), phenformin (Sigma‐Aldrich, St. Louis, MO, USA), and A769662 (Tocris Bioscience, Bristol, UK) for 24 h for monitoring FANCD2 activation. For the pretreatment experiment, cells were treated with Compound C (Tocris Bioscience) 1 h before AICAR treatment.

### Western blotting

Cells were washed in phosphate‐buffered saline (PBS) and lysed in lysis buffer (50 mm Tris/Cl, pH 7.4, 150 mm NaCl, 0.3% Igepal CA‐630, 0.2% Triton X‐100, 10 mm NaF, 1 mm sodium orthovanadate, and protease inhibitors). Lysates were cleared by centrifugation and supernatants were obtained. Equal amounts of lysate were resolved on 3–8% NuPAGE Tris‐acetate gel (Invitrogen, Carlsbad, CA, USA). Resolved proteins were electro‐transferred onto nitrocellulose membrane (GE Healthcare Europe, Freiburg, Germany) and detected by immunoblotting with antibodies to FANCD2 (NB100‐182; Novus Biologicals, Littleton, CO, USA), phopho‐AMPKα (#2535, Cell Signaling Technology, Danvers, MA, USA), AMPKα (#2532, Cell Signaling Technology), FANCA (A301‐980A, Bethyl Laboratories, Montgomery, TX, USA), p53 (OP43, Calbiochem), p21^Cip1^ (#2947, Cell Signaling Technology), PARP‐1 (#9542, Cell Signaling Technology), and p15^Ink4B^ (#4822, Cell Signaling Technology). Protein bands were visualized with the ECL Prime kit (GE Healthcare UK, Little Chalfont, UK).

### Confocal microscopy

Confocal microscopy was performed as described previously 19. Cells grown on coverslips and treated with AICAR for 24 h were fixed in 3.7% paraformaldehyde in PBS for 20 min, permeabilized with 0.2% Triton X‐100 in PBS for 10 min and blocked with 1% bovine serum albumin in PBS for 1 h. Fixed cells were incubated overnight with anti‐FANCD2 (Novus Biologicals), washed in PBS supplemented with 0.2% Tween 20 (PBST) and incubated with Alexa 488‐conjugated donkey anti‐rabbit antibody for 3 h. Finally, the cells were washed with PBST, stained with 4′,6‐diamidino‐2‐phenylindole (DAPI), mounted on glass slides and observed with a Zeiss Axiovert LSM780 microscope (Carl Zeiss, Oberkochen, Germany).

### Cell proliferation/survival assay

For 3‐(4,5‐dimethylthiazol‐2‐yl)‐2,5‐diphenyltetrazolium bromide (MTT) assays, cells were seeded in 96‐well culture plates at 2 × 10^3^ cells/well and exposed to AICAR at indicated concentrations. Cell survival was evaluated by adding 10 μL of 5 mg·mL^−1^ MTT (Duchefa Biochemie, Haarlem, the Netherlands) to 100 μL of the cell culture medium and incubated for 30 min to 1 h at 37 °C in a CO_2_ incubator. After removal of the medium, formazan crystals were dissolved in dimethly sulfoxide (DMSO), and optical density was measured at 570 nm using SPECTROstar Nano Microplate Reader (BMG Labtech, Ortenberg, Germany).

### siRNA transfection

Caki‐1 cells were transfected with synthetic siRNAs by using the lipofectamine 2000 transfection reagent (Invitrogen), according to the manufacturer's recommendations. In brief, 80 pmol of siRNA duplex and 4 μL of lipofectamine 2000 reagent were added separately to 200 μL of serum‐free medium per well of six‐well plates. After 5 min, the two solutions were mixed and incubated for 20 min at room temperature. The mixture was added to cell monolayers fed fresh medium. All siRNAs targeting AMPKα1 (Hs_PRKAA1_8), FANCA (Hs_FANCA_5 and Hs_FANCA_6), and FANCD2 (Hs_FANCD2_2 and Hs_FANCD2_4) were purchased from Qiagen (Hilden, Germany). Negative control siRNA was purchased from Bioneer (Daejeon, Korea).

### Cell cycle analysis

Cells were treated with 0.25 mm AICAR for 2 days and then harvested and fixed in 90% methanol. After washing with PBS, cells were resuspended in PBS containing 25 μg·mL^−1^ propidium iodide plus 77 μg·mL^−1^ RNase A (Sigma‐Aldrich). Flow cytometry was performed using a BD LSRFortessa™ cell analyzer (BD Biosciences, Franklin Lakes, NJ, USA).

## Results

### FANCD2 is activated by AMPK‐activating AICAR treatments

**Figure 1:**
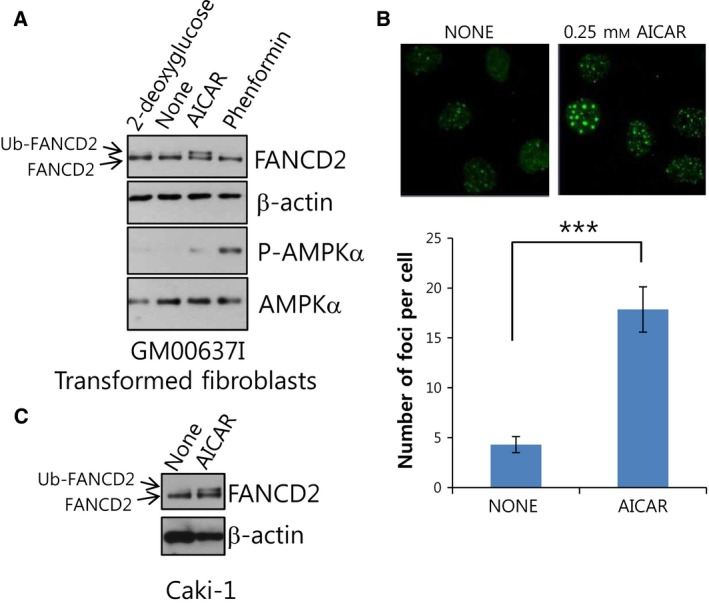
AMPK‐activating AICAR treatment activates FANCD2, a pivotal molecule of Fanconi anemia DNA damage signaling pathway. (A) AICAR treatment induces FANCD2 monoubiquitination in transformed normal fibroblasts (GM00637I). GM00637I cells were treated with 1 mm 2‐deoxyglucose, 0.25 mM AICAR, or 1 mm phenformin for 24 h. Lysates were subjected to western blotting with anti‐FANCD2, phospho‐AMPKα1 (T172), and AMPKα and β‐actin. In FANCD2 blots, the position of monoubiquitinated FANCD2 (Ub‐FANCD2) is indicated by an arrow. (B) AICAR treatment induces formation of FANCD2 nuclear foci in GM00637I fibroblasts. Cells grown on coverslips in 12‐well plates were treated with 0.25 mm AICAR for 24 h. Cells were immunostained with FANCD2 antibody and Alexa 488‐conjugated anti‐rabbit secondary antibody. FANCD2 foci were visualized by confocal microscopy. Representative images are shown at the top. The number of foci per cell was counted and plotted for ≥ 20 cells (bottom panel). The values represent the mean ± SEM (Student's *t*‐test, ***P < 0.001). (C) AICAR treatment induces FANCD2 monoubiquitination in Caki‐1 cells. Caki‐1 cells were treated with 0.25 mm AICAR for 24 h and monoubiquitination of FANCD2 was monitored as in (A).

For investigating whether treatment with chemicals modulating the cellular energy status affect the FA pathway, we monitored mobility‐shifted monoubiquitinated FANCD2 bands on immunoblots (Ub‐FANCD2 in Fig. 1A). FANCD2 monoubiquitination was induced neither by treatment of the transformed fibroblast cell line, GM00637I with 2‐deoxyglucose (2‐DG), which inhibits glycolysis, nor by phenformin, which inhibits complex 1 of the electron transport chain. In contrast, treatments with AICAR, an AMPK activator, increased robust Ub‐FANCD2 bands (Fig. 1A). It is well established that monoubiquitinated FANCD2 is rich in chromatin fractions and forms nuclear foci, which can be readily detected by immunofluorescence confocal microscopy. Confocal microscopy clearly showed FANCD2 foci in AICAR‐treated GM00637I fibroblasts (Fig. 1B). Furthermore, robust FANCD2 monoubiquitination was also observed after AICAR treatment of Caki‐1 renal cell carcinoma cells (Fig. 1C). These results indicate that AICAR, a well‐known AMPK‐activating energy disruptor, is able to induce FANCD2 activation, which typically occurs in a DNA damage signaling process in response to DNA interstrand crosslink (ICL) lesions.

### AICAR‐induced FANCD2 activation is dependent on AMPK

**Figure 2:**
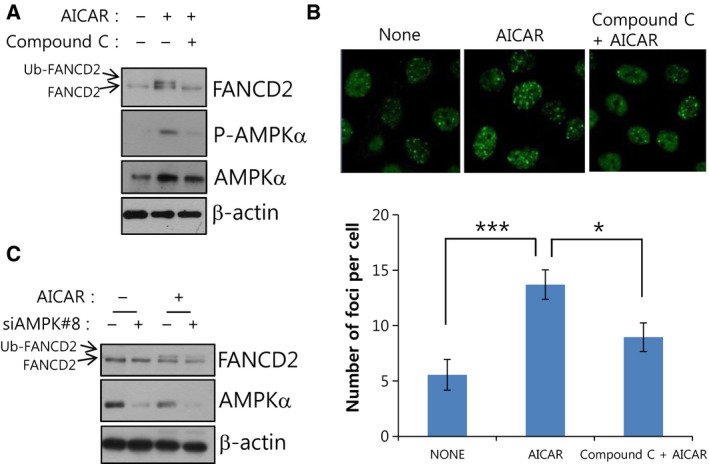
AICAR‐induced FANCD2 monoubiquitination is dependent on AMPK. (A) Inhibitor of AMPK blocks AICAR‐induced FANCD2 monoubiquitination in GM00637I normal fibroblasts. Cells were pretreated with 5 μm of Compound C (an AMPK inhibitor) 1 h before treatment with 0.25 mm AICAR for 24 h. Cell lysates were subjected to immunoblotting with FANCD2 to visualize monoubiquitinated FANCD2 (Ub‐FANCD2). (B) AMPK inhibitor abrogates AICAR‐induced FANCD2 nuclear foci formation in GM00637I fibroblasts. GM006387I cells grown on coverslips were pretreated with 5 μm Compound C 1 h prior to 0.25 mm AICAR treatment for 24 h. FANCD2 foci were visualized by immunofluorescence staining and confocal microscopy. Representative images are shown at the top. The number of foci per cell was counted and plotted for ≥ 20 cells (bottom panel). The values represent the mean ± SEM (Student's *t*‐test, *P < 0.05; ***P < 0.001). (C) Knockdown of AMPKα1 inhibits AICAR‐induced FANCD2 monoubiquitination in Caki‐1 cells. Caki‐1 cells were transfected with siRNAs (siControl or siAMPK#8) and after 48 h, AICAR was treated for 24 h. FANCD2 monoubiquitination was monitored by immunoblotting.

We have previously reported that AMPK is involved in FANCD2 activation after ICL‐inducing mitomycin C (MMC) treatments 15. As AICAR is a well‐known activator of AMPK, we checked whether AICAR‐induced FANCD2 activation is dependent on AMPK. First, we pretreated transformed fibroblast GM00637I cells with Compound C, an AMPK inhibitor, 1 h before AICAR treatment. Western blotting results revealed that AICAR‐induced FANCD2 monoubiquitination was abolished in Compound C‐pretreated cells (lane 3 in Fig. 2A). AICAR‐induced FANCD2 foci formation also decreased in transformed fibroblasts pretreated with Compound C (Fig. 2B). Furthermore, AMPKα1 knockdown in Caki‐1 cells also resulted in a similar inhibition of AICAR‐induced FANCD2 monoubiquitination (lane 4 in Fig. 2C), substantiating the requirement of AMPK in AICAR‐induced FANCD2 activation.

### AICAR‐induced FANCD2 activation requires the FANCA protein

**Figure 3:**
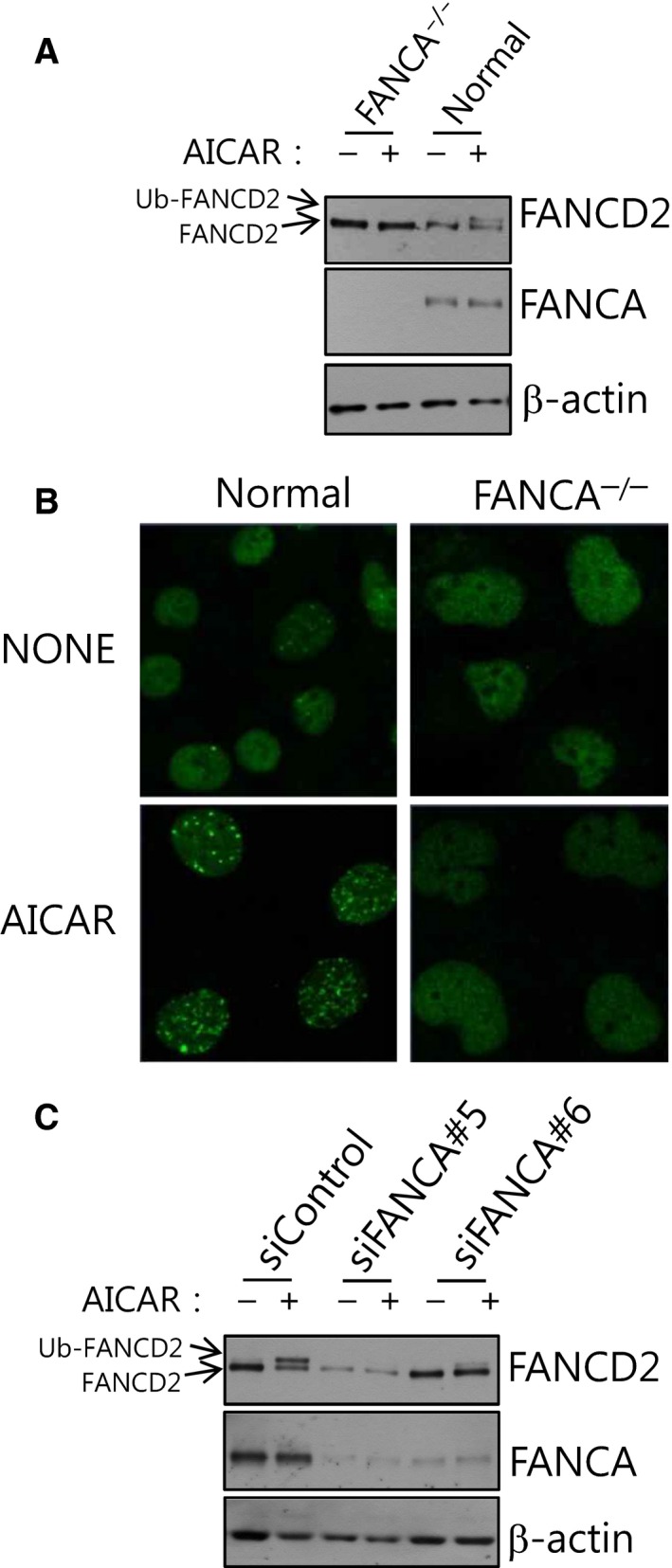
AICAR‐induced FANCD2 monoubiquitination requires FANCA. (A) AICAR‐induced FANCD2 monoubiquitination does not occur in transformed FANCA ^−/−^ fibroblasts originated from a patient with defective FANCA. Transformed FANCA ^−/−^ (GM06914B) or normal fibroblasts (GM00637I) were treated with 0.25 mm AICAR for 24 h, and monoubiquitinated FANCD2 (Ub‐FANCD2) was visualized by immunoblotting. (B) AICAR‐induced FANCD2 nuclear foci formation is abrogated in FANCA ^−/−^ fibroblasts. FANCD2 nuclear foci were visualized by immunofluorescence staining and confocal microscopy. (C) Knockdown of FANCA abolishes FANCD2 monoubiquitination after AICAR treatment in Caki‐1 cells. Caki‐1 cells were transfected with FANCA‐targeting siRNAs (siFANCA#5 or siFANCA#6), treated with AICAR and subjected to FANCD2 immunoblotting.

FANCD2 monoubiquitination upon DNA damage occurs through the activity of the FA core complex, which comprises eight FANC proteins including FANCA and FANCL 5. Without FANCA, the MMC‐induced FANCD2 monoubiquitination is severely inhibited. To see whether AICAR‐induced FANCD2 activation has similar requirements, we monitored FANCD2 monoubiquitination in transformed FANCA^−/−^ fibroblast cells (GM0614B) derived from an FA patient with defective FANCA. It was revealed that AICAR‐induced FANCD2 monoubiquitination (Fig. 3A) and nuclear foci formation (Fig. 3B) were completely abolished in GM0614B. Furthermore, when FANCA was knocked down by FANCA‐specific siRNAs (siFANCA#5 and siFANCA#6) in Caki‐1 cells, FANCD2 monoubiquitination was inhibited in AICAR‐treated FANCA knockdown cells (Fig. 3C). These results indicate that the FA core complex may be responsible for the AICAR‐induced FANCD2 monoubiquitination.

### FANCD2 loss or knockdown affects AICAR‐induced apoptosis and cell cycle progression

**Figure 4:**
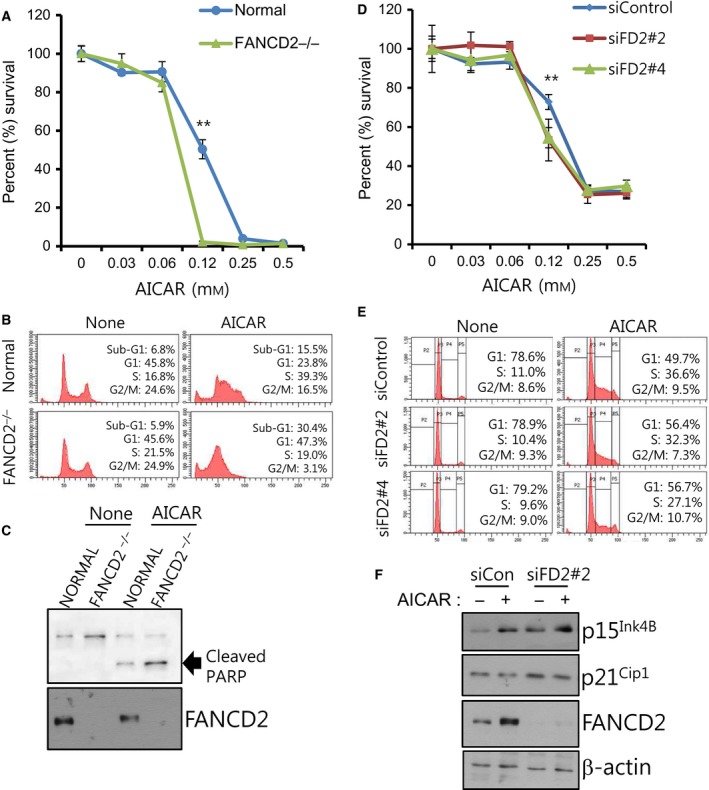
AICAR‐induced apoptosis and cell cycle arrest are affected by FANCD2 repression. (A) Effects of FANCD2 knockout on cell proliferation/survival after AICAR treatment in transformed fibroblasts. MTT assay was performed for normal and FANCD2^−/−^ fibroblasts treated with the indicated concentrations of AICAR for 3 days. The percent (%) survival was calculated for comparison with untreated cells. A representative graph from three independent experiments is shown. The values represent the mean ± SD (Student's *t*‐test, **P < 0.01). (B) Cell cycle analysis of normal and FANCD2^−/−^ fibroblasts treated with AICAR. Cells were treated with 0.25 mm AICAR for 2 days, and flow cytometric analysis was performed after propidium iodide staining. A representative data from three independent experiments are shown. The percentages of cells in the sub‐G1 population are shown inside each graph. (C) Effects of FANCD2 loss on AICAR‐induced PARP cleavage. Transformed fibroblasts were treated with 0.25 mm AICAR for 2 days, and cleaved PARP was detected via immunoblotting. (D) Effects of FANCD2 knockdown on cell proliferation/survival after AICAR treatment in Caki‐1 cells. Caki‐1 cells were transfected with control siRNA (siControl) or FANCD2‐targeting siRNAs (siFD2#2 or siFD2#4), and MTT assay was performed. The percentage survival was calculated for comparison with untreated cells. A representative graph from three independent experiments is shown. The values represent the mean ± SD (Student's *t*‐test, **P < 0.01). (E) Effects of FANCD2 knockdown on cell cycle distribution of AICAR‐treated Caki‐1 cells. Cells were transfected with siControl, siFD2#2, or siFD2#4. Cell cycle analysis was performed as in (B). The percentages of cells at G1 and S phases are shown inside each graph. (F) Effects of FANCD2 knockdown on levels of cell cycle regulators. Caki‐1 cells were transfected with control siRNA (siCon) or FANCD2 siRNA (siFD2#2) and treated with AICAR for 24 h. The levels of p15^Ink4B^ and p21^Cip1^ were evaluated by immunoblotting.

AICAR treatment significantly inhibits proliferation of various cancer cell lines, and this inhibition involves induction of cell cycle inhibitor proteins such as p21, p27, and p53, and S‐phase arrest 20. In the CaSKi cervical cancer cell line, AICAR induced S‐phase arrest and promoted apoptosis 21. To test whether FANCD2 has roles in cellular physiological response to AICAR, we monitored the consequences of FANCD2 loss or knockdown at the cellular level. First, when the cell proliferation/survival after AICAR treatments was evaluated via the MTT assay in transformed fibroblasts established from an FA patient with defective FANCD2, FANCD2^−/−^ fibroblast (GM16633A) were found to be more sensitive to AICAR treatments when compared with normal fibroblasts (Fig. 4A). The cell cycle analysis of AICAR‐treated fibroblasts revealed increases in the percentages of both sub‐G1 and G1 populations in FANCD2^−/−^ fibroblasts, compared with those of normal fibroblasts (Fig. 4B). Consistent with these results, cleaved PARP, a marker of apoptosis, also increased in the FANCD2^−/−^ fibroblasts (Fig. 4C). These results indicate that complete knockout of FANCD2 in transformed fibroblasts enhanced cytotoxicity of AICAR, possibly through apoptosis augmentation.

In case of Caki‐1 renal cell carcinoma cells, cell proliferation/survival decreased after FANCD2 knockdown with FANCD2 siRNAs (siFD2#2 or siFD2#4) compared with control siRNA‐transfected cells (siControl) at the AICAR concentration of 0.12 mm in MTT assay (Fig. 4D). However, AICAR treatment of Caki‐1 cells did not increase the percentages of cells in sub‐G1 population but changed the cell cycle distribution (Fig. 4E). After AICAR treatment of siControl‐transfected Caki‐1 cells, the percentages of cells in G1 phase decreased and those in S phase increased. In case of cells transfected with siFD2#2 or siFD2#4, fewer cells were in S phase and more cells in G1 phase in comparison with siControl‐transfected cells (Fig. 4E). This might be due to enhanced G1/S cell cycle checkpoint activation. Supporting this possibility, FANCD2 knockdown in Caki‐1 cells increased the levels of p15^Ink4B^ and p21^Cip1^, which are cell cycle inhibitors in G1/S phase (Fig. 4F). These results indicate that FANCD2 has certain roles in regulating the cell cycle progression after AICAR treatment in Caki‐1 cells.

## Discussion

Based on our previous results showing a functional association between AMPK and FANC proteins, in this study we investigated whether energy‐disrupting treatments activate the FA pathway, which is typically activated upon DNA ICLs. We found that AMPK‐activating AICAR could robustly activate FANCD2, a pivotal protein molecule of the FA pathway (Fig. 1). AICAR‐induced FANCD2 activation needs AMPK activity (Fig. 2), implying that functional interaction between AMPK and FANC proteins also play a certain role in AICAR‐induced FANCD2 monoubiquitination. FANCA, which are critical for FANCD2 activation upon DNA damage, was also required for AICAR‐induced FANCD2 activation (Fig. 3). From these results, we propose that FA pathway could be activated by AICAR, an AMPK‐activating energy‐disrupting chemical, and that this event requires the activities of AMPK and FANCA.

Among energy‐disrupting treatments, only AICAR treatment activated FANCD2. Phenformin induced AMPK phosphorylation but not FANCD2 monoubiquitination (Fig. 1A), implying that AMPK activation is not sufficient for FANCD2 activation and that other factors may be involved. Biguanides including phenformin and metformin indirectly activate AMPK by inhibiting complex 1 of the mitochondrial respiratory chain and increasing cellular AMP level 22. In contrast, AICAR directly activates AMPK but does not disturb AMP level 17. It is notable that AICAR inhibits anabolic processes, especially lipid and cholesterol synthesis via inhibition of acetyl‐CoA carboxylase and 3‐hydroxy‐3‐methylglutaryl‐CoA reductase, respectively 17, 23. Further study is needed to identify the full requirements for AICAR‐induced FANCD2 monoubiquitination.

Regarding the mechanism of FANCD2 activation by AICAR, the chemicals may not have a direct effect but may cause indirect activation by oxidative stress induced by aberrant energy metabolism. AICAR generates reactive oxygen species (ROS) 24. Thus, AICAR‐induced FANCD2 monoubiquitination might be the consequence of oxidative DNA damage elicited by ROS. To test this hypothesis, we monitored ROS generation by AICAR in transformed fibroblasts and Caki‐1 cells by 2′,7′‐dichlorodihydrofluorescein diacetate (DCF‐DA) staining experiments. The results showed that AICAR did not induce ROS generation in both cell lines (Fig. S1A and data not shown). Furthermore, AICAR‐induced monoubiquitination was not affected by pretreatments with ROS‐scavenging N‐acetyl cysteine (Fig. S1B), thus excluding the possibility of ROS involvement in AICAR‐induced FANCD2 activation.

Whether monoubiquitinated FANCD2 has a certain role in cytoplasm remains to be elucidated. A previous study showed that monoubiquitinated FANCD2 is detectable only in chromatin fractions, not in soluble fractions 25, and that FANCD2 with a defective nuclear localization signal (NLS) was not monoubiquitinated upon MMC treatment, raising the argument that FANCD2 monoubiquitination occurs principally in the nucleus. However, FANCD2 monoubiquitination might occur in the cytoplasm, as the FA core complex is indeed detected in cytoplasm with a molecular mass of 500–600 kDa 26.

A close connection between DNA damage response and cellular metabolism regulation is emerging. Notably, ATM, a key regulator in response to DNA double‐strand breaks, participates in the oxidative stress response and regulates mitochondrial function 27. A recent report on the functions of FANC proteins in the process of mitophagy, a special autophagy mechanism for mitochondria 14, is considered a milestone for novel functions of FANC proteins. The results in this study are expected to broaden our understanding of FANCD2 function and add to the existing knowledge of cellular energy metabolism regulation.

Regarding the role of FANCD2 in cellular effects of AICAR, FANCD2 loss resulted in more apoptosis induction (Fig. 4B,C). In contrast, knockdown of FANCD2 in Caki‐1 cells increased levels of p21^Cip1^ and p15^Ink4B^, cell cycle inhibitors (Fig. 4F) and enhanced G1/S arrest (Fig. 4E). Consistent with our study, a previous study showed that inhibition of FANCD2 gene expression induced p53 phosphorylation, p21 induction, and G1 cell cycle arrest in the MG‐63 osteosarcoma cell line 28. Our study also points out that consequences of FANCD2 repression might be different depending on cell contexts.

Augmentation of AICAR‐induced cell cycle arrest and cell death by FANCD2 repression might shed new light on the development of anticancer therapeutics. Although AICAR has been reported to have anticancer effects for many cancer cell lines, high concentrations in millimolar range are needed for the effect 17. Our data might raise the feasibility of FANCD2 inhibition as an AICAR‐sensitizing strategy. With a further mechanistic study, it might be possible to develop strategies for enhancing AICAR efficacy.

## Conclusions

This study shows that AMPK‐activating AICAR treatment induces activation of FANCD2, which is a key molecule of FA DNA damage pathway. We have also presented data supporting the involvement of FANCD2 in AICAR‐induced cell cycle arrest and apoptosis.

## Author contributions

CHL conceived and designed the project, MJC, HC, DWJ, and CHL performed experiments and acquired data, SK, YNK, SYK, and CHL analyzed and interpreted data, and MJC, SK, YNK, SYK, and CHL wrote the paper.

## Supporting information


**Fig. S1.** AICAR‐induced FANCD2 monoubiquitination does not involve generation of reactive oxygen species (ROS).
