# Supplementary material for: Fanconi anemia protein FANCD2 is activated by AICAR, a modulator of AMPK and cellular energy metabolism
Source: FEBS Open Bio. 2017 Jan 9;7(2):284–92. doi: 10.1002/2211-5463.12185 (PMC5292659; doi:10.1002/2211-5463.12185)
Supplement: Supplementary file 1 — Fig. S1. AICAR‐induced FANCD2 monoubiquitination does not involve generation of reactive oxygen species (ROS). [file FEB4-7-284-s001.pdf]

A

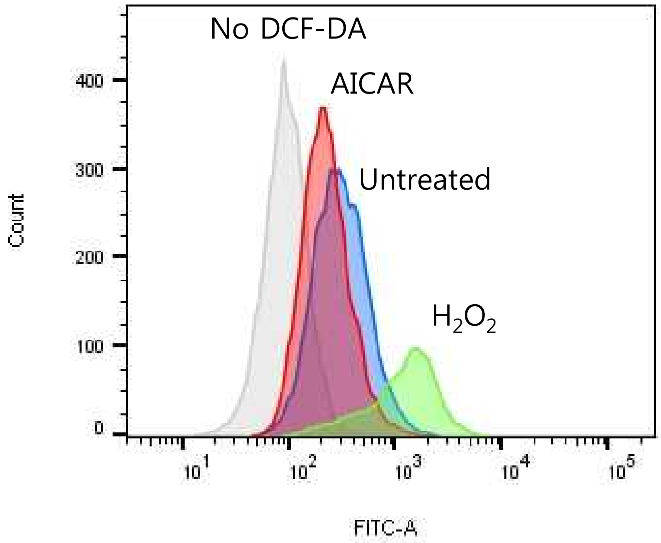

B

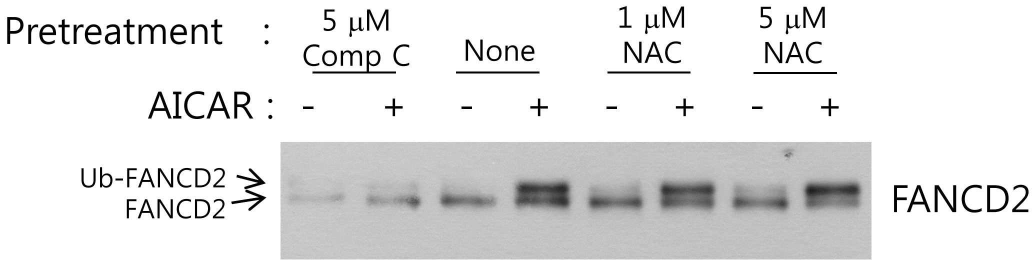

Supplementary Figure 1. AICAR-induced FANCD2 monoubiquitination does not involve generation of reactive oxygen species (ROS). *A. ROS is not generated after AICAR treatments in normal fibroblasts.* Cells were treated with 0.25 mM AICAR for 24 h, stained with DCF-DA and analysed by flow cytometry. Data from AICAR-treated sample (red) are superimposed with untreated, control (blue) and unstained control (grey). In the right graph, H_2O_2-treated samples were included as a positive control. *B. Pretreatment with ROS scavenger, N-acetylcystein (NAC), does not affect the AICAR-induced FANCD2 monoubiquitination.* Caki-1 cells were pretreated with Compound C (Comp C) or NAC 1 h before AICAR treatment for 24 h. Cell lysates were subjected to immunoblotting with FANCD2.
